# Exploring Linkages between Taxonomic and Functional Profiles of the Human Microbiome

**DOI:** 10.1128/mSystems.00163-17

**Published:** 2018-03-27

**Authors:** Morgan G. I. Langille

**Affiliations:** a Department of Pharmacology, Dalhousie University, Halifax, Nova Scotia, Canada

**Keywords:** 16S, function, human microbiome, metagenomics, taxonomy

## Abstract

Microbiome studies typically focus on characterizing the taxonomic and functional profiles of the microbes within a community. Functional profiling is generally thought to be superior to taxonomic profiling for investigating human-microbe interactions, but there are several limitations and challenges to existing approaches. This Perspective discusses the current sequencing and bioinformatic methods for producing taxonomic and functional profiles, recent studies utilizing and comparing these technologies, and the existing challenges and limitations of these data. In addition, functional versus taxonomic conservation across the population is questioned, while future research that focuses on investigating the taxonomic diversity of microbial functions is proposed.

## Abstract (precis)

Microbiome studies typically focus on characterizing the taxonomic and functional profiles of the microbes within a community. Functional profiling is generally thought to be superior to taxonomic profiling for investigating human-microbe interactions, but there are several limitations and challenges to existing approaches.

## PERSPECTIVE

The characterization of microbiomes can be undertaken using various technologies. The most prevalent are 16S rRNA gene surveys (16S) and metagenome studies. Although the 16S approach is less expensive, it is routinely seen as inferior to metagenomics since the former is limited to identifying only the taxa that can be amplified by the chosen set of “universal” primers, thus biasing particular clades of bacteria and archaea and often missing all of microbial eukaryotes and viruses. Further, metagenomics provides insights into the functional capabilities of the microbiome by profiling relative abundances of genes within the microbial community, while 16S is primarily limited to describing taxonomic changes. However, for many host-associated microbiome environments, 16S is the only feasible profiling technique due to host contamination swamping the majority of microbial signal from metagenomic techniques. Therefore, 16S is likely to be a commonly used method, even as sequencing costs continue to decrease.

Predictive methods that leverage large reference genome databases and ancestral state reconstruction methods such as PICRUSt (1) provide insight into the functional repertoire based on 16S profiles. These predictive methods provide functional hypotheses that, like many other technologies, can be validated with specific sequencing primers or through metabolomic analysis. PICRUSt 2.0 (https://github.com/picrust/picrust2) is being actively developed, with a final release being planned in 2018, and provides several new features in comparison to its predecessor. These include predictions based on any 16S sequence originating from an operational taxonomic unit (OTU) clustering approach or from an amplicon sequence variant approach (2), which provides resolution to the level of single nucleotide differences in the 16S rRNA gene instead of the previous 97% sequence identity cutoff commonly used in OTUs. In addition, PICRUSt 2.0 will be based on over 39,000 genomes (a nearly 10-fold increase in reference genomes) and will provide predictions that integrate with the MetaCyc functional framework (3). The accuracy of predicting the eukaryotic functional proportion of the microbiome based on 18S rRNA gene profiles is also being tested and validated. A major limitation of both metagenomics and PICRUSt inferences is that they depend on accurate gene annotations. Previous research has shown that microbial gene annotations are notoriously inaccurate, making biological interpretations of microbiome community function uncertain (4). In addition, these genes may not be transcribed or translated, limiting the impact of their annotated function. Conclusions about microbiome function derived either from metagenomics or from PICRUSt should be treated as hypotheses that require further in-depth validation through functional assays.

Overall, it is intuitive and generally thought that function is much more informative than taxonomic information since it is what the organisms do that we care about and not who they are (5). Indeed, it has been noted by several groups that function seems more highly conserved across samples than across taxa, suggesting that function is more resilient across communities than the individual strains that come and go (6). However, comparisons of taxa and function conservation that were more technical and philosophical in nature have suggested that these comparisons are not meaningful due to their being based on completely different scales (7). For example, are metabolic pathways equivalent to taxonomic phyla, genera, or species? The problem is that, although taxa and function are linked, it is impossible to access them on similar scales. As expected, when using comprehensive gene families instead of broadly conserved functional pathways, functional conservation disappears (7). Therefore, describing functions as being more conserved than taxa is an artifact of the methods and databases used in the comparison rather than an actual biological statement. Nonetheless, function provides information on possible mechanisms present between microbes and in microbe-host interactions. These interactions are essential for understanding and modeling microbial communities, especially with respect to the various microbiome-related diseases.

Considering that the majority of disease-microbiome relationships are not defined by a single species but rather by a complex community of microbes, machine learning methods are an obvious approach for understanding these complex data sets. Machine learning methods take as the input sets of features such as abundances of taxa, genes, transcripts, etc., and a training data set for classifications such as those resulting from comparisons of disease patients to healthy controls, responses to treatment, etc., and output a classifier that can be used on a novel data set. Machine learning methods based on metagenomic taxonomic profiles have shown promise for the classification of various diseases such as colorectal cancer, obesity, diabetes, and Crohn’s disease (8). Taxonomic profiles based on 16S data have also been successful in the identification of subtle differences in samples from the gut microbiome of moderately exercised mice (9). It would seem intuitive that inclusion of relative abundances of genes in these models would help improve classification accuracy. Indeed, there are several examples where changes in gene abundances have been more informative than taxonomic differences in examples such as predicting the gut colonization of a strain of *Bifidobacterium longum* (10). However, a previous observation indicated that machine learning models built with 16S taxa were just as accurate as gene abundance profiles predicted using PICRUSt (11). Further, we recently showed that the levels of accuracy of data from metagenomic-based taxonomic profiles and gene abundance profiles were very similar in looking at predicting disease and treatment outcome in pediatric Crohn’s disease (12). These results suggest that there may be major limitations in how we are currently defining and using metagenomic gene abundance profiles. One limitation is that these analyses are based on data in the KEGG database, which is well annotated but not very comprehensive. The relevant functional differences could easily be hidden within those genes that are currently “unknown.” Although genes of unknown function are initially limiting in their biological interpretation, they could provide completely novel insights if they are among the major features used in classifying a microbiome-related disease. Again, inaccuracies in gene annotation, along with not measuring levels of RNA, protein, or metabolites, could also be further hampering the accuracy of classification.

One side benefit of many machine learning methods is that they often provide insight into the most important features for classification. These features provide identification of possible species or functions to be further investigated and are not limited to only those previously characterized. Further, these different types of microbiome features can be combined into a single model to test improved accuracy or to determine the relative levels of importance of different feature types. For example, we recently investigated whether host genetics, metagenomics-based taxon profiles that included viruses and microbial eukaryotes, metagenomic-based gene abundances, 16S taxon profiles, or simply alpha diversity were the most useful for predicting disease and treatment outcome from gut biopsy samples in a pediatric Crohn’s disease cohort (12). We found that, while host genetics and alpha diversity profiles were statistically significant in detecting Crohn’s disease, they had much lower accuracy than 16S genera profiles and was not predictive at all in determining treatment outcome. Further, we found that combining features from different technologies produced a more accurate classifier. Choosing the correct features for machine learning use is an active area of research. Some diseases can be optimally predicted using simple 16S taxonomic profiles, while others require gene abundance information. Other diseases may require going beyond genomics to measuring levels of RNA transcripts, proteins, and metabolites.

Another obvious factor in disease classification from microbiome data is that we need not use taxon information and functional information independently as is currently done in most bioinformatic analyses. Methods that provide linkages between taxa and their respective functions provide much richer and biological relevant information. PICRUSt has the ability to output the taxonomic contributions of its predictions, while Humann2 (http://huttenhower.sph.harvard.edu/humann2) provides links between taxa and functional annotations for metagenomic data. In addition, methods like FishTaco provide methods to link taxonomy to function using statistical and modeling frameworks (13). These methods provide novel information not simply on how the relative abundance of a particular gene changed but on what organism contributed to those changes. For example, the loss of a particular function could be more biologically interesting if that function had been contributed by 10 different species than by a single species. Functions within the human microbiome were recently characterized as core pathways (one body site), multicore pathways (several body sites), and supercore pathways (all body sites), and the taxonomic contributions of each of these were characterized as being different depending on the body site (14). This type of research forms the foundation for further characterizing taxonomic contributions to various functions in the healthy human microbiome. Further research is needed to determine if these functional contributions are consistent across various diseases or whether there exist unique signatures representing correspondences between certain functions and taxa in particular diseases.

A major hurdle that remains for developing microbiome signatures for precision medicine is the limited robustness of classifiers across data sets. Due to the heterogeneity of DNA extraction, library preparation, sequencing, and bioinformatic techniques, meta-analyses are often difficult to conduct. However, despite technical differences, several studies have demonstrated that biological signal does often remain in 16S (15, 16) and metagenomic (8, 17) data sets. These types of studies depend on the public release of microbiome data, and it is in the best interest of all researchers to uphold open data standards (18). In addition, well-established and open standard operating procedures (19) and the development of standards for sample collection and sequencing (20) and of statistical methods for correcting for batch effects (21) will likely make meta-analysis more powerful in the future.

Microbiome studies will continue to rely on existing sequencing technologies. However, the issue of how we leverage this information to move beyond simply listing and cataloging individual microbial taxa and gene abundances is at the forefront of understanding and modeling microbial communities and harnessing the full potential of the human microbiome.
